# Supplementary material for: Preparation, Characterization, and Antioxidant Properties of Selenium-Enriched Tea Peptides
Source: Foods. 2023 Nov 12;12(22):4105. doi: 10.3390/foods12224105 (PMC10670339; doi:10.3390/foods12224105)
Supplement: Supplementary file 1 [file foods-12-04105-s001.zip › foods-2698529-supplementary.pdf]

Table S1 Enzymatic hydrolysis parameters of Se-TPs

Enzyme category	Substrate concentration (%)	pH	Temperature (°C)	Enzyme content (U)
Alcalase	3	9	55	5000
Neutrase	3	7	40	5000
Papain	3	6.5	55	5000
Trypsin	3	7.5	37	5000

Table S2 CCD design factor levels for the Se-TP hydrolysis method optimization

Factors	Levels				
	-2	-1	0	1	-2
pH	5.5	6.25	7	7.75	8.5
T (°C)	45	50	55	60	65
U (U/g of Se-TPs)	500	1250	2000	2750	3500
T (h)	1	2.25	3.5	4.75	6

Table S3 Four-factor, five-level CCD design for the Se-TP hydrolysis method optimization and the investigated response for the experimental results

Number	pH	Temperature (°C)	Enzyme concentration (U/g)	Time (h)	OH• scavenging (%)
	X_1	X_2	X_3	X_4	
1	6.25	50.00	1250.00	2.25	50.21
2	7.75	50.00	1250.00	2.25	57.15
3	6.25	60.00	1250.00	2.25	59.91
4	7.75	60.00	1250.00	2.25	62.55
5	6.25	50.00	2750.00	2.25	54.14
6	7.75	50.00	2750.00	2.25	60.53
7	6.25	60.00	2750.00	2.25	48.50
8	7.75	60.00	2750.00	2.25	53.53
9	6.25	50.00	1250.00	4.75	58.26
10	7.75	50.00	1250.00	4.75	57.40

11	6.25	60.00	1250.00	4.75	59.79
12	7.75	60.00	1250.00	4.75	57.46
13	6.25	50.00	2750.00	4.75	60.53
14	7.75	50.00	2750.00	4.75	61.76
15	6.25	60.00	2750.00	4.75	50.03
16	7.75	60.00	2750.00	4.75	60.22
17	5.50	55.00	2000.00	3.50	58.69
18	8.50	55.00	2000.00	3.50	65.07
19	7.00	45.00	2000.00	3.50	62.25
20	7.00	65.00	2000.00	3.50	60.47
21	7.00	55.00	500.00	3.50	62.25
22	7.00	55.00	3500.00	3.50	55.62
23	7.00	55.00	2000.00	1.00	57.58
24	7.00	55.00	2000.00	6.00	47.82
25-30	7.00	55.00	2000.00	3.50	66.30

Table S4 ANOVA for the response surface quadratic model

Source	Sum of Squares	df	Mean Square	F value	P value
Model	735.75	14	52.55	5.73	0.0009
X_1	73.46	1	73.46	8.00	0.0127
X_2	5.55	1	5.55	0.60	0.4489
X_3	29.85	1	29.85	3.25	0.0915
X_4	0.016	1	0.016	0.0017	0.9676
X_1X_2	0.21	1	0.21	0.023	0.8812
X_1X_3	16.92	1	16.92	1.84	0.1947
X_1X_4	10.19	1	10.19	1.11	0.3087
X_2X_3	106.99	1	106.99	11.66	0.0038
X_2X_4	10.39	1	10.39	1.13	0.3043
X_3X_4	10.19	1	10.19	1.11	0.3087
X_1^2	53.11	1	53.11	5.79	0.0295
X_2^2	63.53	1	63.53	6.92	0.0189
X_3^2	124.22	1	124.22	13.53	0.0022

X_4^2	372.62	1	372.62	40.60	< 0.0001
Residual	137.68	15	9.18		
Lack of fit	137.68	10	13.77		
R-Squared	0.8423				
Pure error	0.000	5	0.000		
Cor total	873.43	29			
